# The efficacy and safety of adding PD-1 blockade to induction chemotherapy and concurrent chemoradiotherapy (IC-CCRT) for locoregionally advanced nasopharyngeal carcinoma: an observational, propensity score-matched analysis

**DOI:** 10.1007/s00262-024-03698-2

**Published:** 2024-05-11

**Authors:** Ya-Nan Jin, Meng-Yun Qiang, Ying Wang, Yu-Jing Lin, Ren-Wei Jiang, Wan-Wei Cao, Wang-Jian Zhang, Si-Yang Wang, Hong-Yu Zhang, Ji-Jin Yao

**Affiliations:** 1 The Cancer Center of the Fifth Affiliated Hospital of Sun Yat-Sen University, Zhuhai, Guangdong Province 519000 China; 2 Hangzhou Institute of Medicine (HIM), Zhejiang Cancer Hospital, Chinese Academy of Sciences, Hangzhou, Zhejiang Province 310022 China; 3 Department of Nuclear Medicine, The Fifth Affiliated Hospital of Sun Yat-sen University, Zhuhai, Guangdong Province 519000 China; 4 Department of Pathology, the Fifth Affiliated Hospital of Sun Yat-sen University, Zhuhai, Guangdong Province 519001 China; 5 Department of Medical Statistics, School of Public Health, Sun Yat-sen University, Guangzhou, Guangdong 510080 China; 6 The Cancer Center of Nasopharyngeal Carcinoma, the Fifth Affiliated Hospital of Sun Yat-sen University, Zhuhai, Guangdong Province 519000 China

**Keywords:** Nasopharyngeal carcinoma, PD-1 blockade, Induction chemotherapy, Efficacy, Toxicity

## Abstract

**Background:**

Despite the success of PD-1 blockade in recurrent/metastatic nasopharyngeal carcinoma (NPC), its effect for locoregionally advanced NPC (LANPC) remains unclear. This study aimed to evaluate the benefit of adding PD-1 blockade to the current standard treatment (gemcitabine and cisplatin IC <induction chemotherapy> plus cisplatin CCRT <concurrent chemoradiotherapy>) for LANPC patients.

**Methods:**

From January 2020 to November 2022, 347 patients with non-metastatic high-risk LANPC (stage III-IVA, excluding T3-4N0) were included. Of the 347 patients, 268 patients were treated with standard treatment (IC-CCRT), and 79 received PD-1 blockade plus IC-CCRT (PD-1 group). For the PD-1 group, PD-1 blockade was given intravenously once every 3 weeks for up to 9 cycles (3 induction and 6 adjuvant). The primary endpoint was disease-free survival (DFS) (i.e. freedom from local/regional/distant failure or death). The propensity score matching (PSM) with the ratio of 1:2 was performed to control confounding factors.

**Results:**

After PSM analysis, 150 patients receiving standard treatment and 75 patients receiving additional PD-1 blockade remained in the current analysis. After three cycles of IC, the PD-1 group had significantly higher rates of complete response (defined as disappearance of all target lesions; 24% vs. 9%; *P* = 0.006) and complete biological response (defined as undetectable cell-free Epstein-Barr virus DNA, cfEBV DNA; 79% vs. 65%; *P* = 0.046) than that in the standard group. And the incidence of grade 3–4 toxicity during IC was 47% in the PD-1 group and 41% in the standard group, with no significant difference (*P* = 0.396). During follow-up period, additional PD-1 blockade to standard treatment improved 3-year DFS from 84 to 95%, with marginal statistical significance (HR, 0.28; 95%CI, 0.06-1.19; *P* = 0.064).

**Conclusion:**

Additiaonl PD-1 blockade to gemcitabine and cisplatin IC and adjuvant treatment results in significant improvement in tumor regression, cfEBV DNA clearance, superior DFS, and comparable toxicity profiles in high-risk LANPC patients.

**Supplementary Information:**

The online version contains supplementary material available at 10.1007/s00262-024-03698-2.

## Introduction

Nasopharyngeal carcinoma (NPC) is highly prevalent in specific geographic regions, with 40% of the world’s cases in southeast China [1]. The control of early-stage NPC (stage I-II) is usually successful; however, the prognosis of patients with locoregionally advanced disease (stage III-IVA) remains unsatisfactory, with nearly 30% patients experiencing disease progression [2, 3]. According to the latest staging system [4], over 70% of NPC patients present with locoregionally advanced disease [5]. Managing advanced disease poses a challenge for clinicians.

According to the 2022 National Comprehensive Cancer Network (NCCN) guidelines, induction chemotherapy (IC) plus concurrent chemoradiotherapy (CCRT) has been listed as a level 1 recommendation for locoregionally advanced NPC (LANPC) [6]. A multicentre phase 3 trial showed that the addition of induction TPF regimen (e.g. docetaxel, cisplatin, and fluorouracil) to CCRT resulted in better 3-year overall survival (OS), distant metastasis-free survival (DMFS), and disease-free survival (DFS) in LANPC with absolute improvements of 6%, 7%, and 8%, respectively [7]. Another multicentre phase 3 trial observed that adding induction gemcitabine and cisplatin (GP) to CCRT significantly improved 3-year OS (with a 4% absolute benefit) and DMFS (with a 7% absolute benefit) [8]. Based mainly on the findings of these two trials, the IC regimens of TPF and GP were consequently recommended as level 1 A evidence for LANPC [6, 9]. However, despite the addition of three cycles of induction TPF or GP regimens to CCRT, nearly 20–30% of LANPC patients still experience disease failure [10, 11]. Therefore, more effective treatment strategies are needed to further improve the prognosis of LANPC.

In recent years, immunotherapy, especially PD-1/PD-L1 inhibitors, has sparked a revolution in the clinical management of cancer. NPC exhibits high levels of PD-L1 expression (over 90% of tumour cells) and abundant infiltration lymphocytes [12–14], suggesting that NPC patients may be potentially suitable for PD-1 blockade therapy. To date, various PD-1 blockades have been evaluated in recurrent or metastatic NPC (R/M-NPC) [15–17]. Particularly, recent phase 3 trials have confirmed the effectiveness of combining GP chemotherapy with PD-1 blockades in R/M NPC [18, 19]. The combination of PD-1 blockades (e.g. toripalimab and camrelizumab) with the GP regimen was subsequently approved for R/M NPC by the Chinese Medical Products Administration in 2021 [9]. However, the efficacy and safety of adding PD-1 blockade to the standard treatment (gemcitabine and cisplatin IC plus cisplatin CCRT) in LANPC remain unclear.

To address the current knowledge gaps, we conducted a real-world study using two different IC therapies consisting of GP with or without PD-1 blockade, followed by CCRT for high-risk LANPC. For the standard group, patients were treated with induction GP regimen followed by CCRT. For the PD-1 group, PD-1 blockade was given intravenously once every 3 weeks for up to 9 cycles (3 induction and 6 adjuvant). The propensity score matching (PSM) analysis was performed to mitigate potential interference resulting from imbalanced patient characteristics between the study groups. This study may serve as a reference for the combination of PD-1 blockade with IC-CCRT in the treatment of LANPC.

## Patients and methods

### Patient selection

**Fig. 1:**
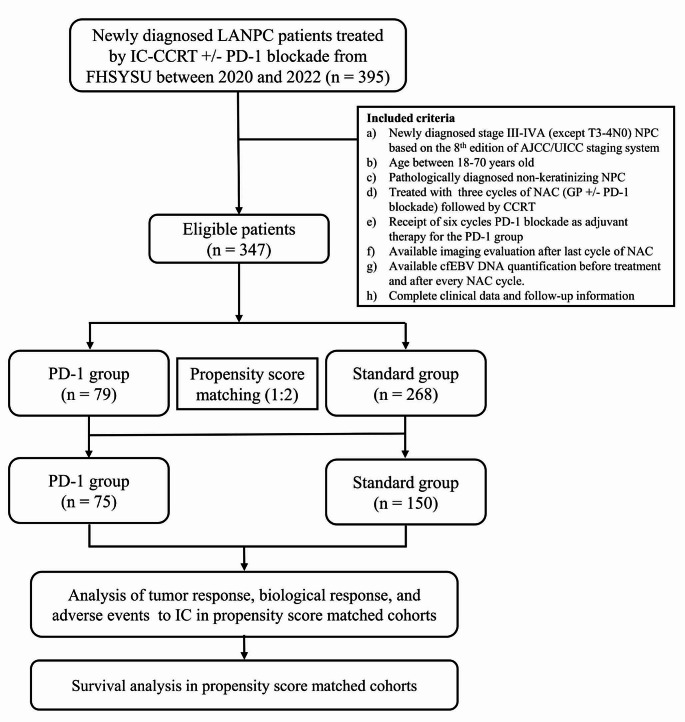
Flow chart of participant inclusion. Abbreviations: LANPC = locoregionally advanced nasopharyngeal carcinoma; IC = induction chemotherapy; CCRT = concurrent chemoradiotherapy; FHSYSU = Fifth Affiliated Hospital of Sun Yat-Sen University; EBV = Epstein–Barr virus; LDH = lactate dehydrogenase; WHO = World Health Organization; Standard group = IC-CCRT; PD-1 group = IC-CCRT plus PD-1 blockade (3 induction and 6 adjuvant)

Medical records of patients diagnosed with non-metastasis NPC at our institution between January 2020 and November 2022 were reviewed. The inclusion criteria were as follows: (a) pathological diagnosis of undifferentiated non-keratinizing carcinomas of the nasopharynx; (b) absence of distant metastases; (c) staging with III–IVA disease (except T3-4N0; according to the 8th AJCC staging system); (d) age 18 years or older with adequate bone marrow, renal, and hepatic functions; (e) receipt of three cycles of IC with GP +/- PD-1 blockade followed by CCRT; (f) available imaging evaluation after the last cycle of IC; (h) available quantification of cell-free EBV DNA (cfEBV DNA) before treatment and after every IC cycle; (i) receipt of six cycles PD-1 blockade as adjuvant therapy for the PD-1 group. The patient inclusion process is illustrated in Fig. 1. This study was approved by the Institutional Review Committee and Ethics Committee of our center (approval number, B2022-016-01).

### Treatment protocol

All patients received radical intensity-modulated radiotherapy (IMRT). Detailed information on IMRT is available in Supplementary Materials. For the standard group, patients were treated with gemcitabine (1 g/m^2^ on days 1,8) and cisplatin (80 mg/m^2^ on day 1) IC plus cisplatin CCRT. For the PD-1 group, besides IC-CCRT, additional PD-1 blockade was given intravenously once every 3 weeks for up to 9 cycles (3 induction and 6 adjuvant). The PD-1 blockades used in the current study included toripalimab, pembrolizumab, tislelizumab, sintilimab, and carelizumab. Patients received toripalimab at a fixed dose of 240 mg or other PD-1 blockades (i.e., pembrolizumab, tislelizumab, sintilimab, or carelizumab) at a fixed dose of 200 mg, all once every 3 weeks. Dose modifications of PD-1 blockade were not permitted. Modification of gemcitabine and cisplatin doses was done according to the locally approved product information. During CCRT, cisplatin (100 mg/m^2^ on days 1, 22, and 43) was administered intravenously.

### Longitudinal cell-free EBV DNA (cfEBV DNA) surveillance

Quantitative reverse transcription polymerase chain reaction was used to determine cfEBV DNA levels at the following time points: 2 weeks before the initiation of IC (pretreatment) and after each cycle of IC (post-IC). Changes in cfEBV DNA from baseline to the first cycle of IC (post-IC_1_) and the last cycle of IC (post-IC_3_) were described. cfEBV DNA = 0 copies/mL was defined as undetectable cfEBV DNA, and cfEBV DNA > 0 copies/ml was defined as detectable cfEBV DNA. Patients with undetectable cfEBV DNA after IC were defined as having a complete biological response (cBR). The quantification of cfEBV DNA is described in Supplementary Materials.

### Assessment of tumor response and toxicity

The Response Evaluation Criteria in Solid Tumors version 1.1 (RECIST 1.1) [20] was used to assess the tumor response to IC. The evaluation of the tumor response rate in patients was mainly based on primary lesions and cervical lymph nodes. In detail, complete response (CR) was defined as the complete disappearance of target lesions. Partial response (PR) was defined as a 30% or greater reduction in the total diameter compared to baseline. Progressive disease (PD) was defined as a 20% or greater increase in the total diameter compared to baseline. Changes that neither reached the level of reduction for PR nor the level of increase for PD were defined as stable disease (SD). Objective response (OR) was defined as complete or partial response confirmed by radiology. The severity of adverse events was graded according to the Common Terminology Criteria for Adverse Events (CTCAE - Version 5.0) [21].

### Immunohistochemistry staining and evaluation

To investigate the relationship between PD-L1 expression and tumor response to the addition of PD-1 blockade, PD-L1 expression on human NPC tissues from the PD-1 group was determined by immunohistochemistry (IHC) staining. First, tumor slices obtained from paraffin-embedded tumor blocks were placed on slides, and then rabbit anti-human PD-L1 monoclonal antibody (1:200; E1L3N, Cell Signaling Technology) was used for staining. All slides were scanned and further analyzed with digital images. The rate of positive PD-L1 expression in digital images (tumor proportion score, TPS) was independently evaluated by three experienced pathologists using the same microscope, and the average TPSs from each examiner per case was recorded. Sections with ≥ 10% tumor staining were considered to have high expression.

### Statistical analysis

Participants were divided into two groups, receiving standard treatment with or without PD-1 blockade: the standard group vs. the PD-1 group. We used the PSM method to select patients who received IC-CCRT, either alone or in combination with PD-1 blockade. PSM is a method used to create sets of similar cases (the standard group) and control sets (the PD-1group) from existing datasets to reduce potential biases in retrospective analysis [22]. The PD-1group and the standard group were matched at a ratio of 1:2 without replacement based on individual covariates. The propensity score for each patient was calculated using logical regression based on the following covariables: age, gender, body mass index (BMI), T stage, N stage, overall stage, and cfEBV DNA. The balance of covariates between the two study groups were examined using a χ2 test.

The primary endpoint of the study was DFS, defined as the time from the date of the first treatment to the any documented disease progression or death form any cause, whichever occurred first. Secondary endpoints included cfEBV DNA clearance, toxicity profile, and OS. OS was defined as the time from the date of the first treatment to the date of death. The rates of tumor response, biological response (cfEBV DNA clearance), and toxicity were compared using the χ² test. Survival rates based on these endpoints (i.e. disease failure and death) were estimated using the Kaplan–Meier method. The changing trend in survival rate among different groups was compared using the log-rank test. Other clinical outcomes and demographic characteristics were summarized descriptively. The statistical tests were double-sided, with a significance level set at 0.05. The analyses were conducted using R version 4.1.0 (https://www.r-pro-ject.org/).

## Results

### Patient characteristics

We identified 347 eligible patients who were diagnosed at our hospital during January 2020 and November 2022 from a specific NPC database. The patient inclusion process is displayed in Fig. 1. In terms of induction regimens, 268 patients received standard treatment, while 79 patients were treated with the addition of PD-1 blockade to standard treatment. Table S1 presents the baseline characteristics of the standard group and PD-1 group. Significant differences were observed between the two groups in terms of BMI (≤ 1.80 m^2^ vs. > 1.80 m^2^) and cfEBV DNA (Undetectable vs. Detectable) (all *P* < 0.05). PSM was performed to minimize the potential interference caused by imbalanced characteristics of the patients in the study group. Subsequently, 150 patients received standard treatment and 75 patients who received the combination of standard treatment and PD-1 blockade remained in the current analysis after PSM. The baseline characteristics of the two groups were well balanced (all *P* > 0.05, as shown in Table 1). The subsequent analyses were conducted based on the matched cohort. Additionally, nearly 50% of patients in the PD-1 group received toripalimab (*n* = 34; 45%), while other PD-1 blockades included pembrolizumab (*n* = 16; 21%), tislelizumab (*n* = 10; 13%), sintilimab (*n* = 9; 12%), and carelizumab (*n* = 6; 8%) in descending order.

**Table 1:** Baseline patient characteristics in the matched cohort

Characteristic		No. (%) of patients by treatment strategy		*P* value^a^
	Entire cohort (n = 225)	GP alone (n = 150)	GP + PD-1 blockade (n = 75)	
Age, years				0.794
≤ 45	103 (45.8)	70 (46.7)	33 (44.0)	
> 45	122 (54.2)	80 (53.3)	42 (56.0)	
Gender				0.999
Male	166 (73.8)	111 (74.0)	55 (73.3)	
Female	59 (26.2)	39 (26.0)	20 (26.7)	
BMI, m^2^				0.101
≤ 1.80	109 (48.4)	79 (52.7)	30 (40.0)	
> 1.80	116 (51.6)	71 (47.3)	45 (60.0)	
T stage^b^				0.865
T1-2	47 (20.9)	32 (21.3)	15 (20.0)	
T3-4	178 (79.1)	118 (78.7)	60 (80.0)	
N stage^b^				0.568
N1	42 (18.7)	25 (16.7)	17 (22.7)	
N2	140 (62.2)	96 (64.0)	44 (58.7)	
N3	43 (19.1)	29 (19.3)	14 (18.7)	
Overall stage^b^				0.886
III	137 (60.9)	92 (61.3)	45 (60.0)	
IVA	88 (39.1)	58 (38.7)	30 (40.0)	
cfEBV DNA, copy/mL				0.876
Undetectable	61 (27.1)	40 (26.7)	21 (28.0)	
Detectable	164 (72.9)	110 (73.3)	54 (72.0)	

*Abbreviations* BMI, body mass index; cfEBV, cell-free Epstein-Barr virus; GP, cisplatin with gemcitabine

^a^*P* values were calculated using the chi-square test or Fisher exact test if indicated

^b^According to the 8th edition of the American Joint Committee on Cancer staging system

### Additional PD-1 blockades and the antitumor activity in the induction GP regimen

**Fig. 2:**
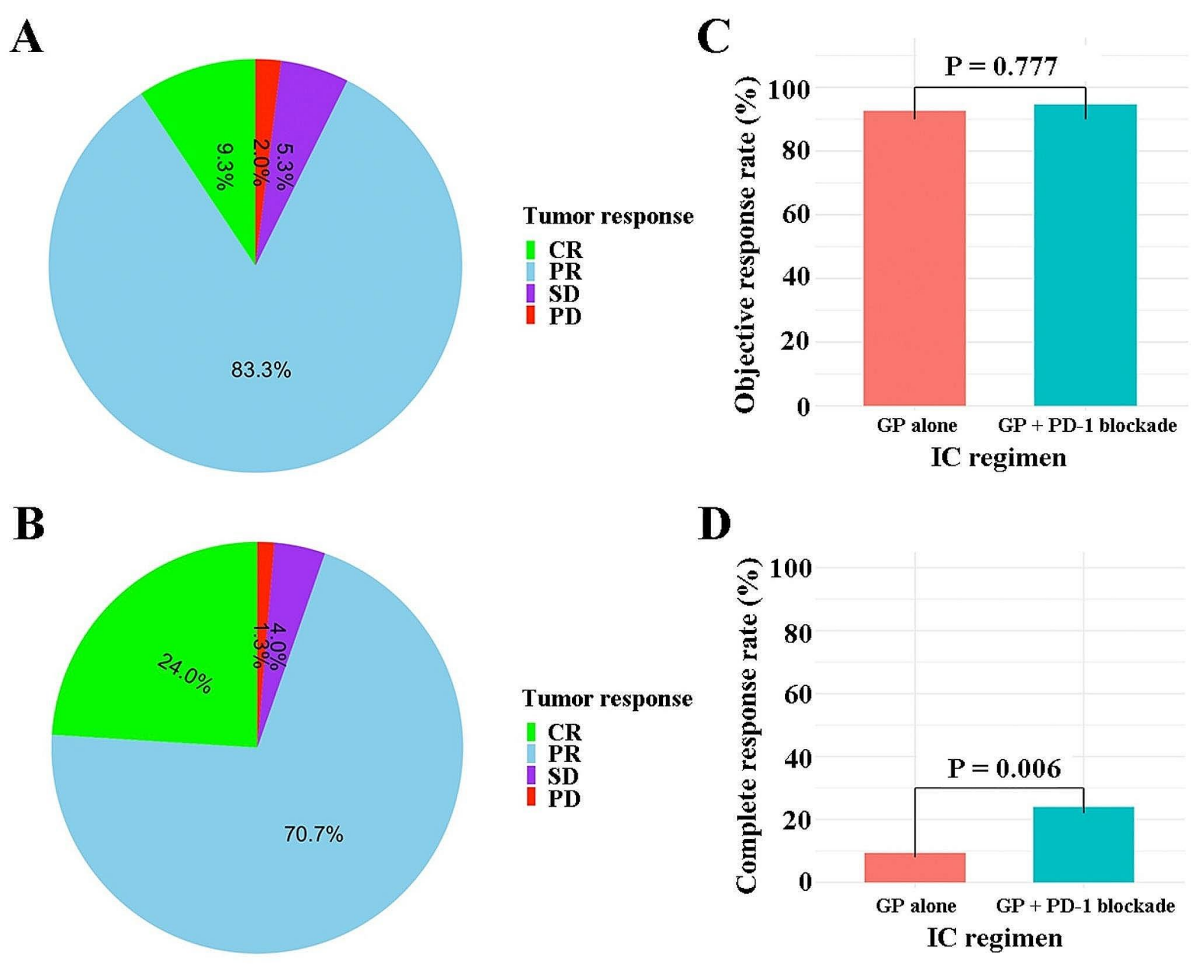
The distribution of tumor response for (**A**) induction GP alone group and (**B**) induction GP + PD-1 blockade group; Comparison of (**C**) objective response and (**D**) complete response between induction GP alone and induction GP + PD-1 blockade. *Abbreviations* CR, complete response; PR, partial response; SD, stable disease; PD, progressive disease; GP, gemcitabine and cisplatin; IC, induction regime

**Fig. 3:**
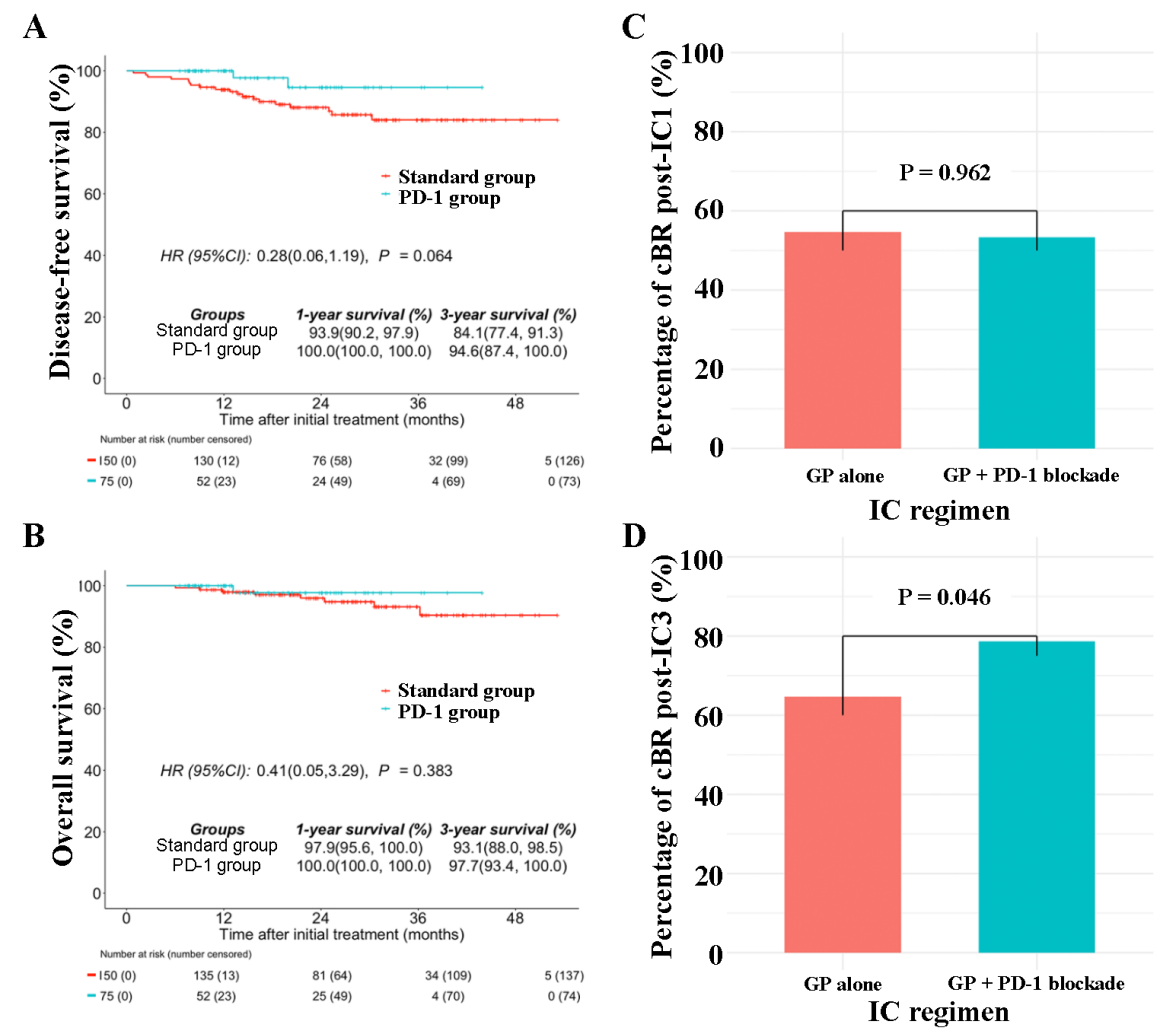
Kaplan–Meier survival curves for (**A**) disease-free survival and (**B**) overall survival between standard group and PD-1 group; The proportion of patients with (**C**) cBR post-IC1 and (**D**) cBR post-IC3 between induction GP alone and induction GP + PD-1 blockade. Abbreviations: cBR, complete biological response; post-IC1, change of cfEBV DNA from baseline to the first cycle of IC; post-IC3, change of cfEBV DNA from baseline to the third cycle of IC; GP, gemcitabine and cisplatin; IC, induction regime; Standard group = IC-CCRT; PD-1 group = IC-CCRT plus PD-1 blockade (3 induction and 6 adjuvant)

In the standard group, 14 patients (9.3%) achieved complete response, 125 (83.3%) had partial response, 8 (5.3%) had stable disease, and 3 (2%) had progressive disease (Fig. 2A). In the PD-1 group, 18 patients (24%) achieved complete response, 53 (70.7%) had partial response, 6 (4%) had stable disease, and 1 (1.3%) had progressive disease (Fig. 2B). Although the objective response rates were similar between both groups (92.7% [139/150] vs. 94.7% [71/75]; *P* = 0.777; Fig. 2C), the proportion of patients achieving complete response in the PD-1 group was significantly higher than that in the standard group (24% [18/75] vs. 9.3% [14/150]; *P* = 0.006; Fig. 2D). In multivariate analyses, the addition of PD-1 blockade was a favorable prognostic factor for complete response (RR [relative risk]: 0.65; 95% CI: 0.31-0.92; *P* = 0.014). To determine whether the addition of PD-1 blockade to the standard treatment provides any additional survival benefit in LANPC, we performed survival comparisons between the two treatment groups. During the median follow-up of 26.5 months, the 3-year DFS rates were 84.1% in the standard group and 94.6% in the PD-1 group (HR, 0.28; 95%CI, 0.06-1.19; *P* = 0.064; Fig. 3A); and the 3-year OS rates were 93.1% and 97.7%, respectively (HR, 0.41; 95%CI, 0.05–3.29; *P* = 0.383; Fig. 3B).

### Association of additional PD-1 blockade with higher cBR

To evaluate the value of additional PD-1 blockade in clearing cfEBV DNA during induction therapy, we excluded 62 patients who had undetectable cfEBV DNA before treatment. Therefore, 107 patients (107/150; 71.3%) in the standard group and 56 patients (56/75; 74.7%) in the PD-1 group remained in the biological response analysis. At post-IC1, the standard group had a comparable rate of cBR to that in the PD-1 group (59 [55.1%] vs. 30 [53.6%]; *P* = 0.962; Fig. 3C). In contrast, the proportion of patients with cBR post-IC3 in the PD-1 group was significantly higher than that in the standard group (44 [78.6%] vs. 70 [65.4%]; *P* = 0.046; Fig. 3D).

### Acute adverse events during induction therapy

We compared the adverse events during induction therapy between the standard group and the PD-1 group (Table 2). We found that among the 61 patients (40.7%) in the standard group, 35 patients (46.7%) in the PD-1 group experienced severe adverse events, with no significant difference between the two groups (*P* = 0.396). The most common severe adverse events included neutropenia (22.7% vs. 25.3%; *P* = 0.739), leucopenia (11.3% vs. 14.7%; *P* = 0.399), vomiting (9.3% vs. 8.0%; *P* = 0.809), and nausea (7.3% vs. 6.7%; *P* = 0.999) in both groups. For immune-mediated adverse events, the PD-1 group also had a higher incidence of grade 3–4 hypothyroidism, thyroiditis, stomatitis, and interstitial pneumonitis compared to the standard group. However, only 5 out of 75 patients (6.7%) in the PD-1 group experienced severe immune-related adverse events, and no significant difference was observed. In addition, there were no reported deaths during the study.

**Table 2:** Acute toxicity during induction chemotherapy

Variable	GP alone (n = 150, %)	GP + PD-1 blockade (n = 75,%)	P value^a^
***Adverse events during induction chemotherapy***			
**Any toxicity**	61 (40.7)	35 (46.7)	0.396
Neutropenia	34 (22.7)	19 (25.3)	0.739
Leucopenia	17 (11.3)	11 (14.7)	0.399
Vomiting	14 (9.3)	6 (8.0)	0.809
Nausea	11 (7.3)	5 (6.7)	0.999
Thrombocytopenia	10 (6.7)	5 (6.7)	0.999
Hepatoxicity	4 (2.7)	4 (5.3)	0.446
Anaemia	3 (2.0)	3 (4.0)	0.403
Nephrotoxicity	2 (1.3)	2 (2.7)	0.602
Allergic reaction	1 (0.7)	1 (1.3)	0.999
***Immune-related adverse events***			
Hypothyroidism	0	2 (2.7)	0.110
Thyroiditis	0	1 (1.3)	0.333
Stomatitis	1 (0.7)	1 (1.3)	0.999
Interstitial pneumonitis	0	1 (1.3)	0.333

*Abbreviations* GP, cisplatin with gemcitabine

^a^*P* values were calculated using the chi-square test or Fisher exact test if indicated

### Correlation between PD-L1 expression and response to induction GP plus PD-1 blockade

**Fig. 4:**
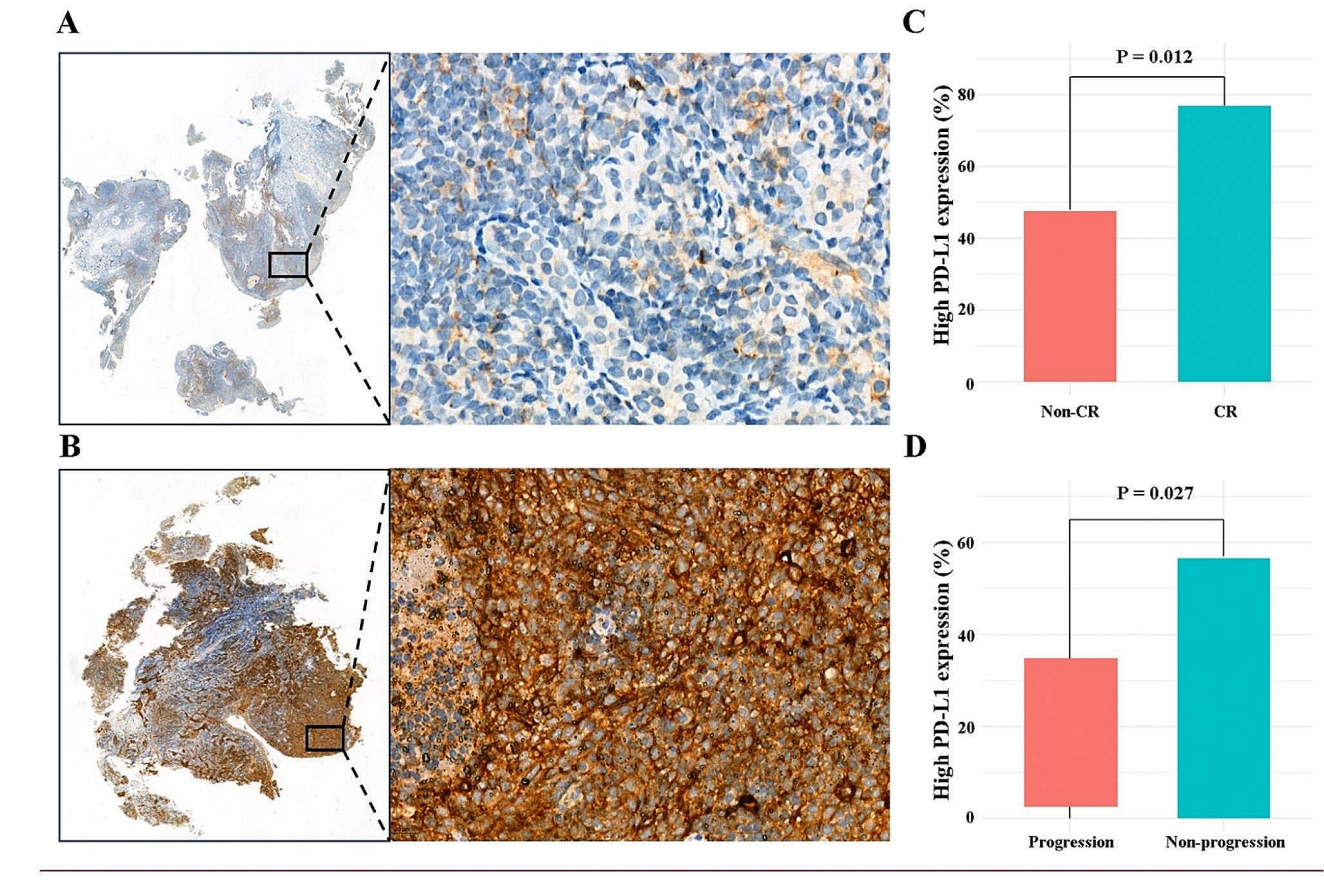
The representative images of PD-L1 positive staining on (**A**) 1 ~ 10% of tumor cells and (**B**) > 10% of tumor cells in immumohistochemical staining; (**C**) the proportion of patients with high PD-L1 expression between responders than non-responders; (**D**) the risk of disease progression between patients with high and low PD-L1 expression

Tumor PD-L1 expression was assessed in 63 patients from the PD-1 group. The IHC analysis showed that all these patients had positive PD-L1 expression on NPC cells (defined as PD-L1 positive staining on ≥ 1% of tumor cells; Fig. 4A). The median PD-L1 expression on NPC cells was 10% (range, 1-80%). Therefore, a uniform cutoff point of 10% (< 10% vs. ≥10%) was chosen to categorize patients into high and low PD-L1 expression groups. Interestingly, the proportion of patients with high PD-L1 expression (defined as PD-L1 positive staining on ≥ 10% of tumor cells; Fig. 4B) was significantly higher in CR patients than in non-CR patients (76.9% vs. 48.8%; *P* = 0.012; Fig. 4C). In addition, we also observed that patients without disease progression were more likely to have high PD-L1 expression compared to those with disease progression (56.7% vs. 33.3%; *P* = 0.027; Fig. 4D).

## Discussion

We conducted this study to evaluate the efficacy and safety of adding PD-1 blockade to IC-CCRT for LANPC patients, and our results suggested that GP induction therapy with PD-1 blockade has a strong effect on tumour shrinkage and tumour marker clearance, with manageable side effects. More importantly, the addition of 9 cycles PD-1 blockade (3 induction and 6 adjuvant) to standard treatment shows an improving trend in DFS, despite the limited follow-up time. This study may provide a reference for the treatment of high-risk LANPC.

Currently, the NCCN Guidelines recommend both TPF and GP as induction regimens for LANPC (Category 1 A) [6]. However, the choice of the optimal induction regimen for LANPC patients remains unclear due to the lack of direct comparisons between TPF and GP regimens. In our study, we evaluated the efficacy and toxicity of combining PD-1 blockade with the GP regimen for high-risk LANPC patients. There are several reasons for selecting GP regimens. Firstly, a retrospective study [23] comparing the efficacy of GP plus CCRT versus TPF plus CCRT for LANPC showed no significant differences in OS and PFS between the two groups at 3 years. However, compared to the GP scheme, the TPF regimen was related to more ≥ 3 grade adverse events. Consistent with the retrospective study, a recent meta-analyses [24] based on eligible trials also reported that the TPF regimen had a higher incidence of toxicity than the GP regimen for LANPC patients. Moreover, they found that patients treated with the GP regimen had better OS and DMFS than those treated with the TPF induction regimen. Secondly, gemcitabine, as a nucleosides mimic, has the potential to consume immunosuppressive cells and activate the antitumor immune response [25, 26]. Given its synergy with immunotherapy and lower incidence of severe adverse events compared to TPF, the GP regimen was chosen as the combination regimen with PD-1 blockade in the present study.

Tumor response rate is an important indicator for the efficacy of therapy. In LANPC patients, a multicenter phase 3 trial found 94.6% of the patients achieved an objective response after three cycles of NAC with the GP regimen, with 10% achieving complete response and 84.5% achieving partial response [8]. Consistent with this phase 3 trial, our study observed comparable rates of objective response (92.7%) and complete response (9.3%) with induction GP alone. Moreover, we found the objective response rates were similar between the induction GP alone and the induction GP + PD-1 blockade. This is reasonable as three cycles of IC with GP already provide an excellent objective response, the the additional benefit of PD-1 blockade on objective response would be limited. However, our results showed that induction GP + PD-1 blockade achieved a higher rate of complete response than induction GP alone. One potential reason for the promising outcomes of adding PD-1 blockade to the GP regimen may be the synergistic effect, as the combination of chemotherapy and PD-1 blockade not only directly kills tumor cells but also has a synergistic effect on eliminating or modulating immune suppressive cells in the tumor microenvironment [27, 28]. We also evaluated the survival benefits of adding PD-1 blockade to standard treatment. Compared to the standard group, the PD-1 group showed superior DFS and OS at 3 years; however, the differences in survival rates were not statistically significant. The failure to demonstrate statistical significance may be due to the limited follow-up time and the small number of patients investigated.

NPC is mainly associated with EBV infection in endemic areas [29]. cfEBV DNA is considered to be a useful biomarker for population screening [30], prognosis prediction [31], and treatment decisions [32]. The predictive and prognostic role of baseline cfEBV DNA in NPC treated with PD-1 blockade is still understudied. Compared with the baseline cfEBV DNA, the dynamic change in cfEBV DNA during treatment could be a more reliable biomarker for prognosis evaluation. Lv et al. [33] reported in a retrospective study of 673 patients that the dynamics of cfEBV DNA clearance during IC were a reliable prognostic predictor for NPC, with early responders showing faster cfEBV DNA clearance during IC and a longer survival time. In this study, the addition of PD-1 blockade to induction GP regimen significantly improved the incidence of cfEBV DNA clearance, and patients with undetectable cfEBV DNA at post-IC3 had a significantly higher rate of complete response. Consistent with our study, the CAPTAIN-1st trial [18] demonstrated that the early clearance of cfEBV DNA was related to a high response rate to the camrelizumab combined GP regimen. Similarly, in a phase I study of camrelizumab combined with GP, NPC patients who had undetectable cfEBV DNA after the first month of therapy had significantly longer DFS than those with detectable cfEBV DNA at baseline [34].

The safety profiles of induction GP regimen observed in our study were consistent with those in other studies [8]. Notably, the most common treatment-related adverse events of induction GP regimen were haematological and gastrointestinal toxicities, which were alleviated after treatment. Although the addition of PD-1 blockade to the induction GP regimen resulted in higher incidences of severe adverse events, the higher incidence of toxicities was mainly attributed to chemotherapy. On the other hand, the incidence of severe immune-related adverse events in the PD-1 group was only 6.7% (5/75), and the main immune-related toxicities were hypothyroidism, which was similar to previous studies [34]. These data indicate that three cycles of induction GP plus PD-1 blockade are generally manageable in LANPC patients.

Identifying biomarkers for predicting tumor response is crucial. Tumor PD-L1 expression is the most commonly used biomarker for predicting the response to PD-1 blockade. In the current study, we found that patients who achieved a complete response after induction GP + PD-1 blockade therapy were more likely to have high levels of PD-L1 expression. Consistent with our findings, a descriptive analysis showed that patients with higher PD-L1 expression levels responded better to nivolumab than those with PD-L1 negative tumors [35]. However, a meta-analysis involving 1836 NPC patients from 15 studies on PD-L1 expression suggested that the expression level of PD-L1 may not be a reliable prognostic biomarker for NPC [36]. Similarly, the phase III JUPITER-02 study evaluated the predictive value of PD-L1 expression in patients with advanced NPC and found a clinical benefit from the combination of PD-1 inhibitor and GP regimen regardless of PD-L1 expressions [19]. In general, the current study did not establish PD-L1 expression as the best biomarker for predicting the efficacy and prognosis of immunotherapy in NPC. Further studies are needed to explore predictive biomarkers derived from peripheral blood or tumor tissues, such as actionable hot spot mutations and tumor mutational burdens, for selecting NPC patients for the combination of standard treatment and PD-1 blockade [15, 35, 37].

One of the main advantages of this study was the head-to-head comparison of LANPC patients who received standard treatment with or without PD-1 blockade using the PSM method in each group. This not only addressed various confounding factors but also mitigated the selection bias related to retrospective analysis. Another advantage was that observational data from real medical records reflected the actual medical process and the health status of patients under real-world conditions. However, some limitations should be noted. The main limitations included the lack of randomization and limited follow-up time. The population that would benefit from the addition of PD-1 blockade to the induction GP regimen still needs to be confirmed in prospective studies with longer follow-up time. Another limitation was the heterogeneity of the PD-1 inhibitors due to the retrospective study design. However, to date, there is no evidence indicating one PD-1 inhibitor is superior, and all PD-1 inhibitors used in our study were approved by the China Food and Drug Administration.

In conclusion, this study provides the first evidence that adding 9 cycles of PD-1 blockade (3 induction and 6 adjuvant) to standard treatment translates into a 10-percentage-point advantage in DFS over standard treatment. Moreover, adding PD-1 inhibitors to the backbone of GP induction therapy could improve the CR rates and cfEBV DNA clearance, with manageable adverse events. Although our research results are not sufficient to change the existing treatment modalities, they may provide confidence and references for future prospective studies on combined chemotherapy and PD-1 inhibitors for the treatment of LANPC.

### Electronic supplementary material

Below is the link to the electronic supplementary material.

Supplementary Material 1

## Data Availability

The datasets generated and/or analyzed during the current study are available from the corresponding author upon reasonable request.
