# Supplementary material for: The efficacy and safety of adding PD-1 blockade to induction chemotherapy and concurrent chemoradiotherapy (IC-CCRT) for locoregionally advanced nasopharyngeal carcinoma: an observational, propensity score-matched analysis
Source: Cancer Immunol Immunother. 2024 May 11;73(7):125. doi: 10.1007/s00262-024-03698-2 (PMC11088572; doi:10.1007/s00262-024-03698-2)
Supplement: Supplementary file 1 — Supplementary Material 1 [file 262_2024_3698_MOESM1_ESM.docx]

**Supplement 1 (Detailed IMRT protocol)**

All patients were treated with radical intensity-modulated radiotherapy (IMRT), while immobilized in the supine position using a thermoplastic head and shoulder mask. Contrast-enhanced planning computed tomography (CT; 3 mm-slice thickness) images from the superior border of the frontal sinus to 2 cm below the sterno-clavicular joint were obtained and transferred to the Monaco treatment planning system (version 3.02; Elekta AB, Stockholm, Sweden). Target volumes and organs at risk (OARs) were delineated on each slice of the CT images, as previously described [1], in agreement with International Commission on Radiation Units and Measurements Reports 62 [2] and 83 [3]. The gross tumor volume (GTV) including primary nasopharyngeal tumor (GTVp) and GTVnd was delineated on the basis of clinical, endoscopic and MRI findings. Gross disease at primary site together with enlarged retropharyngeal lymph nodes was designated GTVp; clinically-involved cervical lymph nodes was designated GTVnd. Two clinical target volumes (CTVs) were delineated according to the GTV: CTV1, high-risk regions encompassing GTVp plus 5-10 mm, including entire nasopharyngeal mucosa and 5 mm submucosal region; and CTV2, low-risk regions containing CTV1 plus 5-10 mm, encompassing sites of microscopic extension and lymphatic regions. The planning target volumes (PTVs), termed PTVp, PTV1, PTV2 and PTVnd, were constructed by expanding the GTVp, CTV1, CTV2 and CTVnd, respectively, by 3 mm; a 3 mm margin was added to the brainstem and spinal cord to generate planning organ at risk volume (PRV). The prescribed doses were 66–72 Gy/28–33 fractions to the planning target volume (PTV) of the primary gross tumour volume (GTVnx), 64–70 Gy/28–33 fractions to the PTV of the GTV of the involved lymph nodes (GTVnd), 60–63 Gy/28–33 fractions to the PTV of the high-risk clinical target volume (CTV1), and 54–56 Gy/28–33 fractions to the PTV of the low-risk clinical target volume (CTV2).

**References**

1. Lai SZ, Li WF, Chen L, Luo W, et al. How does intensity-modulated radiotherapy versus conventional two-dimensional radiotherapy influence the treatment results in nasopharyngeal carcinoma patients? Int J Radiat Oncol Biol Phys. 2011;80(3):661-8.
2. ICRU report. Vol. 62: Prescribing, recording, and reporting photon beam therapy. Maryland: International Commission on Radiation Units and Measurements; 1999.
3. ICRU Report. Vol. 83: Prescribing, Recording, and Reporting Photon-Beam Intensity-Modulated Radiation Therapy (IMRT). Maryland: International Commission on Radiation Units and Measurements; 2010.

**Supplement 2 (Quantification of cfEBV DNA)**

Before treatment, peripheral venous blood (3 mL) was collected from each patient into EDTA-containing tubes and centrifuged at 3000 g for 5 min. Total plasma DNA was extracted using a QIAamp DNA Blood Mini Kit (Qiagen, Hilden, Germany). Fluorescence PCR was carried out using an EBV PCR quantitative diagnostic kit (Da-An Genetic Diagnostic Center, Guangzhou, China) targeting the BamHI-W region of the EBV genome. Data were analyzed using Applied Biosystems 7300 SDS software (Beijing, China). Undetectable cfEBV DNA was defined as 0 copies/mL; and cfEBV DNA > 0 copies/ml was defined as detectable cfEBV DNA.

**Table S1.** Baseline characteristics for the entire group.

|  |  | No. (%) of patients by treatment strategy | |  |
| --- | --- | --- | --- | --- |
| Characteristic | Entire cohort  (n = 347, %) | GP alone  (n = 268, %) | GP + PD-1 blockade  (n = 79, %) | *P* value^a^ |
| Age, years |  |  |  | 0.362 |
| ≤ 45 | 141 (40.6) | 105 (39.2) | 36 (45.6) |  |
| > 45 | 206 (59.4) | 163 (60.8) | 43 (54.4) |  |
| Gender |  |  |  | 0.999 |
| Male | 255 (73.5) | 197 (73.5) | 58 (73.4) |  |
| Female | 92 (26.5) | 71 (26.5) | 21 (26.6) |  |
| BMI, m^2^ |  |  |  | **0.030** |
| ≤ 1.80 | 174 (50.1) | 143 (53.4) | 31 (39.2) |  |
| > 1.80 | 173 (49.9) | 125 (46.6) | 48 (60.8) |  |
| T stage^b^ |  |  |  | 0.876 |
| T1-2 | 71 (20.5) | 54 (20.2) | 17 (21.5) |  |
| T3-4 | 276 (79.5) | 214 (79.8) | 62 (78.5) |  |
| N stage^b^ |  |  |  | 0.985 |
| N1 | 72 (20.8) | 55 (20.5) | 17 (21.5) |  |
| N2 | 209 (60.2) | 162 (60.5) | 47 (59.5) |  |
| N3 | 66 (19.0) | 51 (19.0) | 15 (19.0) |  |
| Overall stage^b^ |  |  |  | 0.526 |
| III | 218 (62.8) | 171 (63.8) | 47 (59.5) |  |
| IVA | 129 (37.2) | 97 (36.2) | 32 (40.5) |  |
| cfEBV DNA, copy/mL |  |  |  | **0.047** |
| Undetectable | 131 (37.8) | 109 (40.7) | 22 (27.8) |  |
| Detectable | 216 (62.2) | 159 (59.3) | 57 (72.2) |  |

**Abbreviation:** BMI, body mass index; cfEBV, cell-free Epstein-Barr virus; GP, cisplatin with gemcitabine.

^a^ *P* values were calculated using the chi-square test or Fisher exact test if indicated.

^b^According to the 8th edition of the American Joint Committee on Cancer staging system.
